# Predicting primary resistance to third-generation EGFR-TKIs in lung adenocarcinoma using a multisource cross-modal transformer model

**DOI:** 10.1038/s41698-026-01420-2

**Published:** 2026-04-17

**Authors:** Yunfan Wang, Ke Min, Li Tao, Jun Jin, Lingling Fan, Xiancheng Yan, QianQian Wang, Dan Wu, Zhiwen Lu, Jian Yang, Chunluan Yuan, Weimin Wang

**Affiliations:** 1 Department of Oncology, The Affiliated Yixing Hospital of Jiangsu University, Yixing, Jiangsu China; 2 Faculty of Medicine, Yangzhou University, Yangzhou, Jiangsu China; 3 Department of Oncology, Lianyungang Clinical College of Nanjing Medical University/The First People’s Hospital of Lianyungang, Lianyungang, Jiangsu China

**Keywords:** Biomarkers, Cancer, Computational biology and bioinformatics, Oncology

## Abstract

The aim of this study was to investigate the utility of a multisource cross-modal Transformer (MC-Trans) model in predicting primary resistance to third-generation epidermal growth factor receptor tyrosine kinase inhibitors (3rd-EGFR-TKIs) in patients with lung adenocarcinoma. A retrospective analysis of clinical and imaging data from 222 lung adenocarcinoma patients treated with 3rd-EGFR-TKIs was conducted. Patients were allocated to a training/validation cohort (*n* = 136) and two external test cohorts (*n* = 34 and *n* = 52). The Table Transformer and a Swin Transformer-based model were employed to extract features from tabular and CT imaging data, respectively, to construct the MC-Trans model. The results demonstrated that MC-Trans exhibited excellent performance in predicting primary resistance, with an ROC-AUC of 0.89, which was significantly superior to those of the unimodal models (tabular model: 0.78; CT model: 0.63). Furthermore, predictions on external Test Cohort 2 revealed that the predictive performance of MC-Trans was comparable to that of a human expert panel. The study also revealed that MC-Trans could predict the risk of disease progression even among patients without primary resistance. In conclusion, MC-Trans may serve as a valuable tool to assist in determining the therapeutic response of lung adenocarcinoma patients to 3rd-EGFR-TKIs. Keywords: Artificial intelligence; Lung Cancer; Multimodal models; Third-generation EGFR-TKIs; Primary Resistance.

## Introduction

Lung cancer is the most prevalent malignancy globally and the leading cause of cancer-related mortality^1^. Activating mutations in the epidermal growth factor receptor (EGFR) are of considerable clinical significance in non-small cell lung cancer (NSCLC), particularly among Chinese patients, where they account for approximately 38% of cases^2^. The two most common EGFR mutation types include exon 19 deletions (ex19Del) and an amino acid substitution in exon 21 (leucine to arginine at codon 858 [L858R])^3^. EGFR tyrosine kinase inhibitors (TKIs) have been shown to confer significant therapeutic benefits to patients with NSCLC harboring EGFR mutations. First- and second-generation TKIs, such as gefitinib, have demonstrated superiority over standard chemotherapy in terms of overall response rate and progression-free survival (PFS)^4,5^. Third-generation EGFR-TKIs (3rd-EGFR-TKIs), exemplified by osimertinib, have shown efficacy in overcoming the T790M mutation, which arises following resistance to first- and second-generation TKIs, and in managing intracranial progression^6^. Nevertheless, approximately 20% of patients with EGFR mutations do not benefit from 3rd-EGFR-TKIs, potentially because of primary resistance^7^. Studies have indicated that mutations in genes such as TP53, EGFR exon 20 insertions (ex20ins), RB1, and KRAS may be associated with primary resistance to 3rd-EGFR-TKIs^8–10^. Currently, there is a lack of definitive clinical or molecular predictive biomarkers to identify patients with primary resistance.

Artificial intelligence (AI) technologies, particularly deep learning, have demonstrated considerable advantages in lung cancer diagnosis and treatment decision-making. Previous studies have explored the potential of AI in predicting lung cancer treatment responses using models such as DeepSurv, CNN, and BigBiGAN^11–13^. However, a significant portion of this research has focused heavily on radiomic features derived from imaging data^11,12^. Although valuable, approaches that rely predominantly on a single data modality (such as imaging) may not fully capture the complex, multifactorial nature of treatment resistance. Recognizing this limitation, a few studies that have incorporated genomic information for predicting TKI efficacy have not combined these with imaging evidence^14^. Some multimodal models have attempted to integrate various data types but are often constrained by limited data sources and incomplete coverage of clinical information^15^. Consequently, the development of deep learning models based on comprehensive patient data to assess responsiveness to EGFR-TKI therapy is urgently needed.

The Transformer model, a novel deep learning architecture, has shown remarkable performance advantages in multiple domains, including natural language processing and computer vision, and has achieved initial progress in medical applications such as biomarker screening and lung cancer prediction^16,17^. In this study, multisource heterogeneous data from multiple centers for patients with lung adenocarcinoma, including clinical information (general information and treatment history), molecular testing results (NGS gene sequencing and body fluid tests), and CT images, were integrated in Table 1. A cross-modal fusion model based on the Transformer architecture was constructed to predict primary resistance to 3rd-EGFR-TKIs. By comparing the model’s predictions with those of an expert panel, this research evaluates the potential value of the multisource cross-modal Transformer (MC-Trans) model in guiding the selection of 3rd-EGFR-TKI therapy for patients with lung adenocarcinoma.

**Table 1:** Feature categorization and variable definitions of multisource cross-modal data in lung adenocarcinoma

Category	Specific Features	Description &Definition
Basic Patient Information	Sex	Categorical (Male / Female)(1/0)
	Age	Continuous (Years)
	Brain metastases; Liver metastasis; Bone metastasis.	Binary (Yes/No)(1/0)
Tumor staging	T-stage	Categorical (T1/T2/T3/T4)(1/2/3/4)
	N-stage	Categorical (N0/N1/N2/N3)(0/1/2/3)
	M-stage	Categorical (M0/M1)(0/1)
	Overall stage	Categorical (III/IV)(3/4)
Treatment	Drug type	Categorical (Osimertinib/ Almonertinib/Firmonertinib)(0/1/2)
	Prior-EGFR-TKIs(1st/2nd); Prior chemotherapy; Prior surgery; Prior radiotherapy; Prior anti-PD-1/PD-L1.	Categorical (Yes/No) (1/0)
Genetic Testing	Number of mutations	Continuous (Count)
	EGFR amplification; EGFR-19del; EGFR-19 mutation; EGFR-19 amplification; EGFR-20 T790M; EGFR-21 L858R; TP53 mutation; PIK3CA mutation; EGFR-20 other mutation; EGFR-21 other mutation; EGFR-18 G719X; KRAS mutation; EZH2 mutation; KEAP1 mutation; BRAF mutation; BRCA1 mutation; LATS1 mutation; HER-2 amplification; ERBB3 amplification; ALK rearrangement; AKT1 mutation; ROS1 rearrangement; MET amplification; ATM mutation; RET mutation; KIT amplification; SMARCA4 mutation; SOX9 mutation; CDK4 amplification; CDK6 amplification; CDK8 mutation; MDM2 amplification; CDKN2A deletion; TGFBR2 mutation; PMS2 amplification; MYC amplification; SRMS mutation; NKX2-1 mutation; NTRK3 fusion; IRS2 mutation; IGF1R mutation.	Categorical (Yes/No) (1/0)
Body fluid testing	White blood cell count (WBC, ×10⁹/L); Monocyte count; Red blood cell count (RBC, ×10¹²/L); Hematocrit (HCT,%); Lymphocyte count(×10⁹/L); Mean platelet volume(fL); Basophil count(×10⁹/L); Eosinophil count(×10⁹/L); Hemoglobin (Hb, g/L); Platelet count (PLT, ×10⁹/L); Neutrophil count(×10⁹/L); Neutrophil-to-lymphocyte ratio (NLR); Albumin(g/L); Albumin/globulin ratio; Gamma-glutamyl transferase (GGT, U/L); Alanine aminotransferase(U/L); Aspartate aminotransferase (AST, U/L); Indirect bilirubin(μmol/L); Alkaline phosphatase(U/L); Prealbumin(mg/L); Globulin(g/L); Lactate dehydrogenase (LDH, U/L); Direct bilirubin(μmol/L); Total bilirubin(μmol/L); Total bile acid (TBA, μmol/L); Total protein (TP, g/L); Beta-2 microglobulin (β2-MG, mg/L); Creatinine(μmol/L); Urea (or Blood Urea Nitrogen – BUN, mmol/L); Uric acid (UA, μmol/L); Carcinoembryonic antigen (CEA, ng/mL); Alpha-fetoprotein(ng/mL); Carbohydrate antigen 125 (CA-125, U/mL); Carbohydrate antigen 19-9 (CA 19-9, U/mL); Activated partial thromboplastin time (APTT,s); International normalized ratio (INR); Thrombin time(s); Prothrombin time(s); Fibrinogen (FIB, g/L).	Continuous
Imaging	Chest CT Scan	Input Modality (Thin-slice non-contrast)

## Results

### Clinical characteristics

**Fig. 1:**
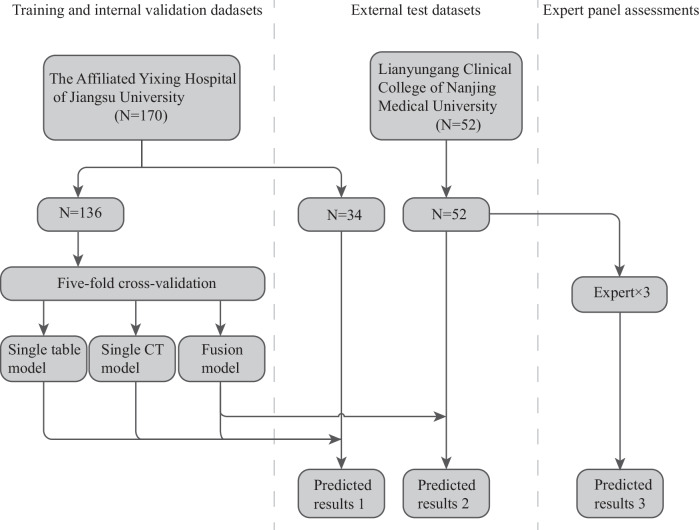
The workflow of this study.

A total of 222 patients were included across the training set, validation set, and two test sets (Fig. 1). The selected clinical characteristics of the patients are presented in Table 2. No significant differences were observed between the data from the training/validation set and external test set 1. Notably, compared with test set 1, test set 2 had a lower proportion of patients who had previously received first- and second-generation EGFR-TKIs, and almonertinib was the predominant drug type. The other characteristics of the test sets were comparable.

**Table 2:** Partial clinical features of all datasets

	Training and validation datasets		Test dataset 1		Test dataset 2	
Ture lable	Class1(*n* = 30)	Class0(*n* = 106)	Class1(*n* = 7)	Class0(*n* = 27)	Class1(*n* = 13)	Class0(*n* = 39)
Sex						
male	11(36.7%)	41(38.7%)	3(42.9%)	8(29.6%)	9(69.2%)	17(43.6%)
female	19(63.3%)	65(61.3%)	4(57.1%)	19(70.4%)	4(30.8%)	22(56.4%)
Age						
avg(SD)	68(8.66)	67(9.04)	67(9.73)	65(11.20)	68(10.30)	66(6.89)
Overall stage						
III	3(10%)	13(12.3%)	0(0%)	3(11.1%)	1(7.7%)	7(17.9%)
IV	27(90%)	93(87.7%)	7(100%)	24(88.9%)	12(92.3%)	32(82.1%)
Prior-1st/2nd EGFR-TKIs						
Yes	16(53.3%)	50(47.2%)	4(57.1%)	13(48.1%)	2(15.4%)	4(10.3%)
No	14(46.7%)	56(52.8%)	3(42.9%)	14(51.9%)	11(84.6%)	35(89.7%)
Prior chemotherapy						
Yes	18(60%)	48(45.3%)	3(42.9%)	11(40.7%)	6(46.2%)	11(28.2%)
No	12(40%)	58(54.7%)	4(57.1%)	16(59.3%)	7(53.8%)	28(71.8%)
Prior surgery						
Yes	7(23.3%)	37(34.9%)	4(57.1%)	16(59.3%)	1(7.7%)	14(35.9%)
No	23(76.7%)	69(65.1%)	3(42.9%)	11(40.7%)	12(92.3%)	25(64.1%)
Prior radiotherapy						
Yes	7(23.3%)	7(6.6%)	2(28.6%)	4(14.8%)	0(0%)	2(5.1%)
No	23(76.7%)	99(93.4%)	5(71.4%)	23(85.2%)	13(100%)	37(94.9%)
Drug type						
Osimertinib	14(46.7%)	53(50%)	3(42.9%)	14(51.9%)	0(0%)	0(0%)
Almonertinib	10(33.3%)	36(34%)	2(28.6%)	9(33.3%)	4(30.8%)	39(100%)
Firmonertinib	6(20%)	17(16%)	2(28.6%)	4(14.8%)	9(69.2%)	0(0%)
Median PFS(Years)	4	16	4	20	4	21

The training and validation datasets were merged because five-fold cross-validation was performed. Class 1: Primary drug resistance. Class 0: Secondary drug resistance. avg: Average value. SD: Standard deviation.

### Fusion model training

**Fig. 2:**
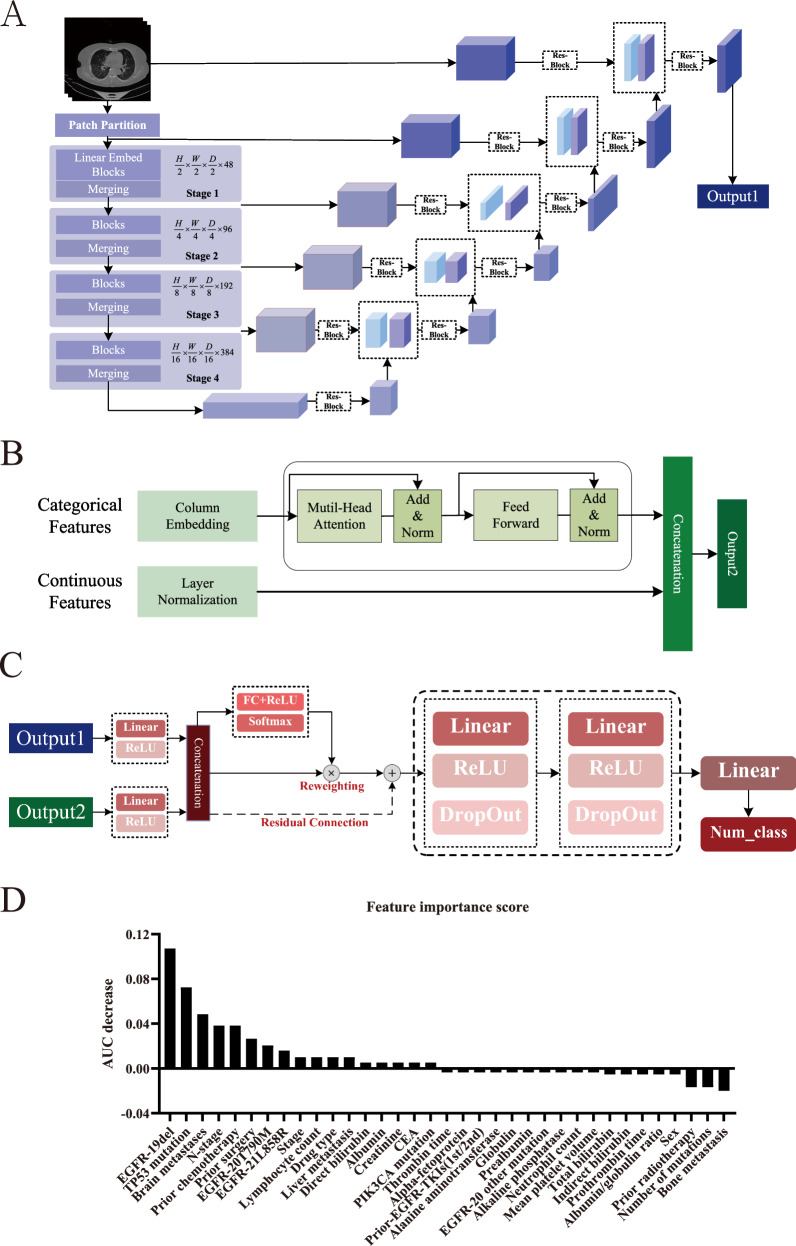
Architecture of the multisource cross-modal transformer (MC-Trans) model and evaluation of clinical feature importance. **A** The 3D Swin Transformer backbone utilized for independent modality feature extraction from chest CT images. **(B)** The TabTransformer network used to process categorical and continuous clinical tabular features. **(C)** The structure of the multimodal fusion module. Feature vectors from both sources (Output 1 and Output 2) are combined and processed through a series of fully connected (Linear) layers, ReLU activation functions, and DropOut modules to generate the final probability prediction for primary resistance. **(D)** Feature importance scores derived from the tabular model, illustrating the impact of different clinical features on the prediction, measured by AUC decrease. EGFR exon 19 deletion, TP53 mutation, and brain metastasis exhibited the most significant effects on the model output. Abbreviations: CT, computed tomography; ReLU, rectified linear unit; AUC, area under the receiver operating characteristic curve.

A multimodal fusion architecture was constructed. First, independent modality feature extraction modules based on the Table Transformer and a Swin Transformer backbone were established (Fig. 2A, B). The feature vectors from both sources were first processed by a gating mechanism to dynamically weight the contributions of each modality. This combined feature vector was then processed through a network comprising a series of fully connected layers (linear) and ReLU activation functions. Specifically, the fused features sequentially underwent linear transformation and ReLU nonlinear activation, with dropout modules introduced between hidden layers to enhance model generalization and prevent overfitting. Finally, the multilayer processed fusion features were passed through a final linear layer to output a probability prediction of primary resistance for the patient. This probability was subsequently used to calculate model performance metrics (Fig. 2C). The models were trained using a 5-fold stratified cross-validation (*k* = 5) setup to maintain class proportions in each fold. To ensure reproducibility, each fold was initialized with a distinct random seed (base seed 42 + fold number). The training was c onducted for 50 epochs for each fold, using a training batch size of 4 and a validation batch size of 8. The optimization strategy involved the AdamW optimizer with an initial learning rate (lr) of 0.01, managed by a StepLR learning rate scheduler (step_size = 10, gamma = 0.1). The binary cross-entropy (BCEWithLogitsLoss) function was applied as the loss function. Key hyperparameters for the 3D Swin branch (processing the 128 × 128 × 128 ROI) included img_size = (128, 128, 128), in_channels=1, feature_size = 48, drop_rate = 0.5, attn_drop_rate = 0.5, and dropout_path_rate = 0.5. Checkpointing (use_checkpoint = True) was enabled to reduce VRAM usage. For the TabTransformer branch, the hyperparameters included dim = 32, depth = 2, heads = 6, attn_dropout = 0.5, and ff_dropout = 0.5. During the training of the tabular model, the weights of different features influencing the output were analysed. EGFR exon 19 deletion, TP53 mutation, and brain metastasis had the greatest effects on the model output (Fig. 2D).

### Comparison between fusion and unimodal models

**Fig. 3:**
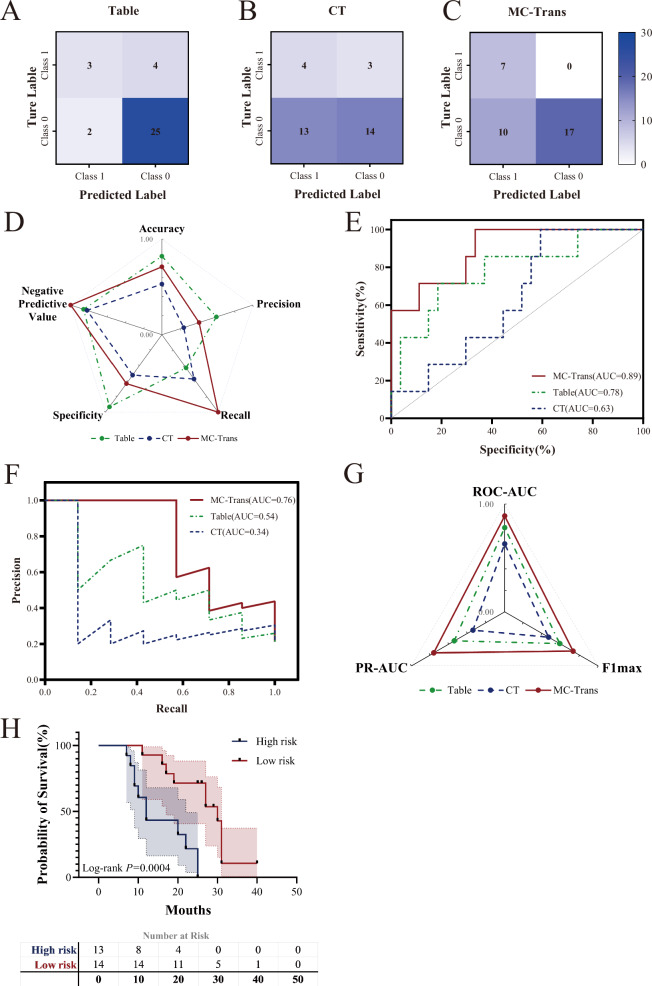
Performance evaluation of the unimodal and MC-Trans fusion models on Test Cohort 1. **A–C** Confusion matrices for the tabular model (A), CT model (B), and MC-Trans model (C), generated using optimized classification thresholds of 0.58, 0.23, and 0.15, respectively. **(D)** Radar chart comparing the models across five performance metrics: accuracy, precision, recall, specificity, and negative predictive value. **(E, F)** Receiver operating characteristic (ROC) curves (E) and precision-recall (PR) curves (F) demonstrating the classification performance of the table, CT, and MC-Trans models. **(G)** Comparison of the three models across overall evaluation dimensions, including ROC-AUC, PR-AUC, and maximal F1 score (F1max). **(H)** Kaplan-Meier survival analysis showing progression-free survival (PFS) in patients with acquired drug resistance (class 0). Patients were stratified into high- and low-risk groups based on the resistance probability predicted by the model (Log-rank *P*=0.0004). Abbreviations: CT computed tomography; ROC receiver operating characteristic; AUC area under the curve; PR precision-recall; PFS progression-free survival.

The performances of the unimodal and MC-Trans fusion models were first evaluated on Test Cohort 1. Owing to the class imbalance in this study (positive samples constituted only 20%), using a fixed threshold (e.g., 0.5) could lead to suboptimal classification accuracy. Therefore, we optimized the classification thresholds using an out-of-fold (OOF) prediction strategy based on fivefold cross-validation. Optimal classification thresholds were determined for each model: 0.58 for the tabular model, 0.23 for the CT model, and 0.15 for the MC-Trans. These optimized thresholds were used for test set evaluation and to generate confusion matrices (Fig. 3A–C). In terms of performance metrics, MC-Trans achieved the highest recall (1.00) and negative predictive value (1.00), whereas the tabular model achieved the highest accuracy (0.82), precision (0.60), and specificity (0.93) (Fig. 3D, Table 3). We further evaluated the models using ROC curves. MC-Trans demonstrated superior classification performance, with an ROC-AUC of 0.89, which was significantly greater than that of the tabular model (0.78, *P* = 0.036, 95% CI [0.0069, 0.2415]) and represented an absolute improvement of 0.26 over that of the CT model (0.63, *P* = 0.092, 95% CI [−0.0476, 0.5238]) (Fig. 3E). Considering the class imbalance in the study data, we also evaluated the PR curves for each model. The MC-Trans performed best, with a PR-AUC of 0.76, which was significantly greater than that of the tabular model (0.54, *P* = 0.028, 95% CI [0.0156, 0.3941]). Although it was also greater than that of the CT model (0.34, *P* = 0.112, 95% CI [−0.1070, 0.7913]), this difference did not reach statistical significance (Fig. 3F). Across multiple evaluation dimensions, including ROC-AUC, PR-AUC, and maximal F1 score, MC-Trans comprehensively outperformed the unimodal models in terms of overall performance (Fig. 3G). Notably, the model also appeared to possess predictive capability for disease progression risk in patients without primary resistance. As detailed in our methods, survival analysis of the acquired resistance group (class 0) revealed that the prognosis of the high-resistance-probability group (as stratified by the model’s output) was significantly worse than that of the low-resistance-probability group (*P* = 0.0004, HR = 3.691, 95% CI [1.335, 10.20]; Fig. 3H).

**Table 3:** Performance comparison of the MC-Trans model, unimodal models, and clinical experts across different cohorts

	Table	CT	MC-Trans (Validation Cohort 1)	MC-Trans (Validation Cohort 2)	Experts (Validation Cohort 2)
Accuracy	0.82	0.53	0.71	0.92	0.88
Precision	0.60	0.24	0.41	0.91	0.88
Recall	0.43	0.57	1.00	0.77	0.62
Specificity	0.93	0.52	0.63	0.97	0.97
Negative Predictive Value	0.86	0.82	1.00	0.93	0.88

### Comparison between MC-trans and human expertise

**Fig. 4:**
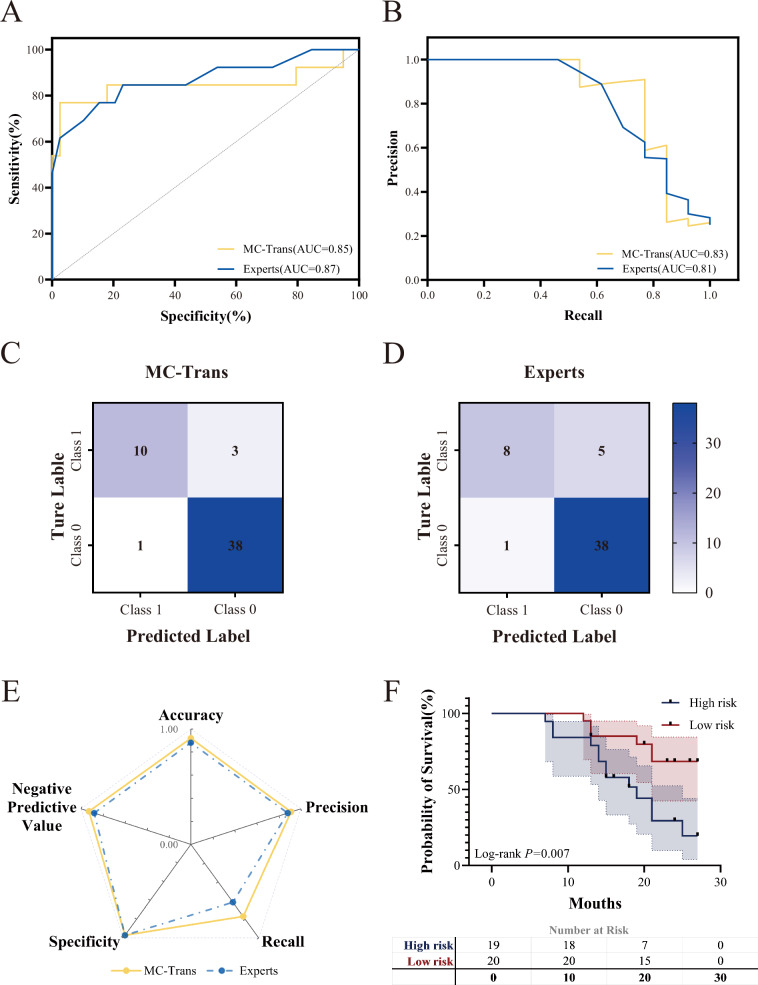
Evaluation of cross-institutional generalizability of the MC-Trans model and performance comparison with clinical experts on Test Cohort 2. **A, B** Receiver operating characteristic (ROC) curves (A) and precision-recall (PR) curves (B) comparing the predictive performance of the MC-Trans model with that of an expert team. **(C, D)** Confusion matrices illustrating the classification results for the MC-Trans model (C) and the clinical experts (D). **(E)** Radar chart comparing the MC-Trans model and the expert team across five evaluation metrics: accuracy, precision, recall, specificity, and negative predictive value. **(F)** Kaplan-Meier survival analysis demonstrating progression-free survival (PFS) differences among patients with acquired drug resistance in the external test set (Test Cohort 2). Patients were stratified into high- and low-risk groups based on the drug resistance probability predicted by the model (Log-rank *P*=0.007). Abbreviations: ROC receiver operating characteristic; AUC area under the curve; PR precision-recall; PFS progression-free survival.

MC-Trans was used to predict outcomes for data in Test Cohort 2, yielding an ROC-AUC of 0.85 (Fig. 4A) and a PR-AUC of 0.83 (Fig. 4B). Numerically, the ROC-AUC for Test Cohort 2 (0.85) was slightly lower than that for Test Cohort 1 (0.89), whereas its PR-AUC (0.83) was greater than that for Test Cohort 1 (0.78). Bootstrap testing revealed that the difference in the ROC-AUC between Test Cohorts 1 and 2 was not statistically significant (*P* = 0.6847, 95% CI [−0.1751, 0.2665]). Similarly, the difference in the PR-AUC between the two cohorts did not reach statistical significance (*P* = 0.7688, 95% CI [−0.4027, 0.2319]). The performance metrics of MC-Trans did not significantly differ between these two independent test sets, and both demonstrated good performance levels. These results indicate that the model has good potential for cross-institutional generalization and can maintain its predictive validity across institutions with different data sources.

We subsequently recruited an expert panel of three experienced medical oncologists and used both MC-Trans and this panel to make predictions on Test Cohort 2. The expert panel achieved an ROC-AUC of 0.87 (Fig. 4A) and a PR-AUC of 0.81 (Fig. 4B). The ROC-AUC of the expert panel was similar to that of MC-Trans (*P* = 0.87, 95% CI [−0.1815, 0.2313]), and their PR-AUC was also similar to that of MC-Trans (*P* = 0.73, 95% CI [−0.3049, 0.2036]). The confusion matrices are shown in Fig. 4C, D, respectively. As optimal classification thresholds could not be determined a priori for the expert panel, thresholds corresponding to the maximal F1 score were used for both. The specificity of MC-Trans is the same as that of the expert group, and its Accuracy, Precision, Recall, and Negative Predictive Value were all slightly higher than those of the expert group. (Fig. 4E, Table 3). These findings suggest that the ability of MC-Trans to identify primary resistance to 3rd-EGFR-TKIs may be comparable to that of experienced medical oncologists. Furthermore, this ability to predict disease progression risk was also observed in Test Cohort 2. Survival analysis, which was conducted as described in the Methods section, revealed that the prognosis of the high-resistance-probability group was significantly worse than that of the low-resistance-probability group (*P* = 0.0074, HR = 3.298, 95% CI [1.315, 8.269], Fig. 4F). These findings further corroborate the conclusion that primary resistance to 3rd-EGFR-TKIs is likely influenced by complex factors.

## Discussion

Third-generation EGFR-TKIs have become the first-line standard of care for EGFR mutation-positive advanced NSCLC^18^. However, previous studies have indicated that approximately 20% of patients exhibit primary resistance to these agents. The therapeutic response of patients to 3rd-EGFR-TKIs directly impacts their prognosis, leading to substantial disparities in survival benefits. Therefore, accurately predicting treatment efficacy before initiation is a pressing clinical need. Existing research has explored various predictive methods, with radiomics-based models being commonly used to predict EGFR-TKI efficacy^19^. Concurrently, some studies have attempted to integrate laboratory test parameters and pharmacokinetic data to enhance the predictive performance^20^. However, these approaches largely overlook the potential unique information and overlapping effects among different data modalities. In contrast, multimodal models have been shown to effectively predict the efficacy of anti-HER2 therapy in gastric cancer and the prognosis of resectable lung adenocarcinoma^21,22^, demonstrating their advantages in integrating heterogeneous data.

This study successfully developed and validated a multimodal deep learning model, MC-Trans, designed to predict primary resistance to 3rd-EGFR-TKIs in patients with lung adenocarcinoma. The model integrates clinical tabular data comprising 98 variables with CT imaging information. MC-Trans effectively captures synergistic and complementary information between different data modalities, thereby overcoming the biases and limitations associated with single-source information. Evaluation results from both the internal validation set and external independent test sets indicated promising predictive efficacy for MC-Trans. In a head-to-head comparison with a human expert panel, the model’s performance was also comparable. These results are encouraging; however, they must be interpreted in the context of the study’s limitations, particularly the sample size of the test sets. These findings suggest that MC-Trans holds considerable potential for clinical application and is promising for assisting physicians in formulating more precise and personalized treatment strategies, thereby optimizing clinical management pathways for patients with EGFR mutation-positive lung adenocarcinoma.

To position our results in the broader research context, we compare our model’s performance with the literature in Table 4. Although direct comparisons are challenging due to the use of different prediction tasks (e.g., primary resistance vs. PFS), our MC-Trans model (ROC-AUC 0.85–0.89) shows highly competitive performance. It notably outperforms models based on unimodal data^23^ or simpler tabular data^20^ for TKI response. Crucially, many existing studies focus on predicting PFS^11,24^, which is a prognostic marker. In contrast, our model focuses on predicting primary drug resistance—a unique and pressing clinical challenge. Through its strong performance on this specific task, this study demonstrates a more direct and practical value in guiding initial treatment decisions.

**Table 4:** Performance comparison of the proposed model with previously published studies

Study	Model / Key Input Data	Prediction Task	Key Performance Metric (Test Set)
This Study	Multimodal (Chest CT + 98 Tabular Features)	Primary Resistance (to 3rd-Gen)	ROC-AUC: 0.89/0.85; PR-AUC: 0.76/0.83
You S, et al. ^25^	Unimodal (Brain MRI)	TKI Therapy Response	ROC-AUC: 0.726/0.728
Lu CF, et al. ^11^	Multimodal (5 clinical and 5 radiomic features)	PFS (at 12/24 mouths)	ROC-AUC: 0.77 (12 m), 0.86 (24 m)
Lou N, et al. ^22^	Tabular (36 Features)	Therapy Response (BoR) (T790M + )	ROC-AUC: 0.728/0.732
Liu Y, et al. ^26^	Unimodal (CT)	PFS (at 9/12 mouths)	ROC-AUC: 0.858 (9 m), 0.873 (12 m)

Notably, in this study, the unimodal model using only CT imaging data yielded relatively low ROC-AUC and PR-AUC values. However, when CT imaging data were fused with clinical tabular data, the AUC values of MC-Trans significantly improved compared with those of the unimodal models. This phenomenon suggests a strong complementarity between CT imaging and tabular data for this study cohort, highlighting the critical role of multimodal fusion strategies in enhancing model performance in deep learning research aimed at predicting EGFR-TKI resistance. Furthermore, we observed that MC-Trans also possessed a certain ability to predict disease progression risk in patients without primary resistance. Notably, PFS data were not directly used for model training. Nevertheless, in the test set, the predicted probability values output by MC-Trans could still effectively differentiate the relative duration of PFS among patients with acquired resistance. These findings suggest that some patients may experience shortened PFS because only single or a few high-risk factors are present, whereas others may exhibit primary resistance due to the cumulative effects of multiple high-risk factors. This phenomenon indirectly reveals the complex biological mechanisms underlying primary resistance to 3rd-EGFR-TKIs. Our results highlight a key trade-off: the unimodal tabular model tended to minimize false positives (FPs), whereas the MC-Trans model excelled at minimizing false negatives (FNs), achieving high recall (1.0 in Test Cohort 1). This distinction is clinically critical. A false negative (predicting a patient as nonresistant when they are, in fact, resistant) is the more severe error, as it leads to administering ineffective therapy, wasting critical time, and delaying the switch to an appropriate alternative treatment. False positives (incorrectly predicting resistance when there is no actual resistance) may lead to the noninitiation or delay of third-generation EGFR-TKI treatment but can avoid the harm of ineffective treatment. Therefore, the high recall (sensitivity) of the MC-Trans model is its primary clinical advantage, positioning it as a strong screening tool to prevent ineffective therapy and guide clinicians towards better early treatment strategies.

In terms of clinical translation, MC-Trans demonstrated robust generalization capabilities in an external, cross-center dataset, and its predictive accuracy was favorable compared to that of the human expert panel. Based on these findings, we propose that for patients predicted by the model to have a high risk of primary resistance and who also face a significant financial burden, clinicians might prudently consider immunotherapy or combination chemotherapy as alternative or preferential treatment options over 3rd-EGFR-TKIs.

Furthermore, this work provides a crucial foundation for the development of more advanced AI agents in oncology. By demonstrating the successful fusion of heterogeneous data (clinical, genomic, and imaging), our MC-Trans model can serve as a core predictive engine. Such an engine is essential for future AI agents designed to move beyond simple prediction and offer comprehensive, interactive decision support, thereby accelerating the translation of complex multimodal data into personalized treatment strategies for patients.

Although MC-Trans has achieved encouraging preliminary results, this study has several limitations. First, this study has several limitations regarding its sample size. The overall cohort of 222 patients was relatively limited. More importantly, the proportion of patients with primary resistance (the positive class) was low, constituting only 20% of the cohort. This significant class imbalance and the resulting small number of positive samples could compromise model stability and generalizability, particularly in identifying this resistant subgroup. To mitigate the impact of this imbalance, we employed specific methodological strategies. We prioritized the precision-recall area under the curve (PR-AUC) as a key performance indicator, as it is more informative than the ROC-AUC in imbalanced datasets. Our model achieved a strong PR-AUC (0.76), demonstrating its capability despite the data skew. Additionally, we optimized the classification thresholds based on an out-of-fold strategy rather than using a naive 0.5 threshold. Nevertheless, this imbalance remains a key limitation. Furthermore, the training data were primarily sourced from a single medical center, which, combined with the class imbalance, may further restrict the broader generalizability of the model. Future prospective studies with larger, multicentre cohorts that include a greater number of positive cases are necessary to validate and enhance the robustness of the model. Second, there is room for expansion in terms of the data dimensions included in the model; for instance, important multiomics data such as digital pathology images and positron emission tomography-computed tomography (PET-CT) scans were not incorporated. Third, significant heterogeneity existed between the study cohorts, particularly in Test Cohort 2. As shown in the results, Test Cohort 2 had significant differences in treatment regimens (i.e., a predominance of almonertinib and furmonertinib instead of osimertinib) and a markedly lower proportion of prior TKI exposure than the other cohorts did. These discrepancies likely reflect evolving clinical practices and regional factors. For instance, the training/validation cohort included patients from 2018, when many had received 1st/2nd-generation TKIs prior to 3rd-generation agents, whereas Test Cohort 2 (mainly 2023–2024) reflects the more recent standard-of-care of using 3rd-generation TKIs as the first-line treatment. Furthermore, regional insurance policies and drug pricing in the Chinese market may have driven the preference for almonertinib or furmonertinib. Although these tests cross-institutional generalization, they also introduce substantial confounding bias. It is possible that the model’s high performance partly reflects these differences in treatment strategy or cohort characteristics rather than purely biological resistance signals. This has critical implications for the clinical application of the model: its performance may degrade when it is applied to new datasets from other regions (e.g., populations primarily treated with osimertinib) or those with different characteristic distributions. Therefore, further external validation, calibration, and local fine-tuning are imperative before MC-Trans can be generalized to new clinical settings. Finally, to promote MC-Trans for broader clinical application, it is imperative to include data from more medical centers for prospective validation and external calibration, along with more detailed comparative studies involving clinicians. Future work will focus on addressing these limitations and further optimizing the model to achieve more accurate and personalized predictions of primary resistance to 3rd-EGFR-TKIs.

The MC-Trans model constructed in this study offers a promising strategy for leveraging artificial intelligence to improve the accuracy of predicting primary resistance to 3rd-EGFR-TKIs. By effectively fusing multisource, cross-modal data and harnessing the powerful feature extraction capabilities of the Transformer architecture, this model achieves accurate prediction of patient treatment response prior to therapy initiation. The successful development of MC-Trans not only robustly demonstrates the immense potential of artificial intelligence and multimodal data fusion in precision medicine but also presents a new course for future oncological research.

## Methods

### Study design and participants

This multicentre retrospective observational study enrolled patients with stage III/IV lung adenocarcinoma who received 3rd-EGFR-TKI therapy at two independent institutions (Yixing Hospital Affiliated with Jiangsu University and Lianyungang Hospital Affiliated with Xuzhou Medical University) between January 1, 2018, and April 1, 2025. A total of 222 patients with stage III/IV lung adenocarcinoma treated with 3rd-EGFR-TKIs were included. The primary resistance group was defined as patients whose best overall response was progressive disease (PD) or stable disease (SD) lasting ≤6 months. Patients in the acquired resistance group were defined as those whose best overall response was a partial response (PR)/complete response (CR) or whose disease duration was >6 months^25^.

The study workflow is illustrated in Fig. 1. Among the 170 patients from Yixing Hospital Affiliated with Jiangsu University, 80% (*n* = 136) were allocated to the training and validation cohorts via stratified sampling, with the remaining 20% (*n* = 34) assigned to external Test Cohort 1. The 52 patients from Lianyungang Hospital Affiliated with Xuzhou Medical University were assigned to external Test Cohort 2. Test Cohort 1 was used to evaluate the performance of the MC-Trans model. Comparisons between Test Cohorts 1 and 2 were performed to preliminarily assess the cross-institutional generalization capability of MC-Trans. The predictions for Test Cohort 2 were also compared with those of an expert panel to evaluate the concordance between MC-Trans and human expert judgment.

This study received approval from the ethics committees of all the participating institutions. Given the retrospective nature of the analysis, the requirement for informed consent was waived.

Patient selection involved several exclusion criteria. First, patients were excluded if they had a follow-up duration ≤6 months, the presence of other concurrent malignancies, or an absence of visible pulmonary lesions. Furthermore, to ensure the quality and integrity of our high-dimensional dataset (98 features) and to avoid potential biases from data imputation, we adopted a strict complete-case analysis approach. This meant that any patient with missing data for the variables used in the model was also excluded from the study. This data-driven exclusion applied to patients who lacked complete data from NGS genomic profiling, essential pretreatment laboratory parameters, baseline CT imaging, or a clear prior treatment history. This combined selection strategy resulted in the final cohort of 222 patients, whose values were not missing across all 98 features.

For this cohort, general characteristics, tumor staging information, NGS-based genomic profiling data, and laboratory fluid test data were collected from the medical records of each hospital. Thin-slice noncontrast chest CT scans were retrieved from the Picture Archiving and Communication System (PACS). These scans were acquired using various CT scanners, including models from SIEMENS (SOMATOM Definition Flash, Sensation 16, SOMATOM go. Top, SOMATOM Drive), GE MEDICAL SYSTEMS (Optima CT620), and UIH (uCT 790, uCT 860). The acquisition protocol included a noncontrast scan with a resolution of ≥512 × 512 and a slice thickness of ≤2 mm. All the data used in this study were collected within two weeks before or after the first oral administration of 3rd-EGFR-TKIs. The detailed data are listed in Table 1.

PFS, PD, SD, PR, and CR in this study were defined according to the RECIST 1.1 criteria^26^. Patients without documented disease progression or those lost to follow-up were censored at the date of their last follow-up.

### Input data preparation

All the input data underwent further cleaning and standardization. Patients were first classified into the primary resistance group (class 1) and the acquired resistance group (class 0). All the data, excluding the CT images, were entered into a tabular format. Categorical variables (e.g., sex, gene mutation status, and prior chemotherapy history) were processed using label encoding, while continuous variables (e.g., white blood cell count and hemoglobin level) were standardized using Z-score normalization (Table 1). For CT image preparation, radiologist-based segmentation was not needed. The raw DICOM images were first converted into 3D images in the Nrrd format. To standardize CT images of varying dimensions, isotropic resampling and trilinear interpolation were subsequently applied to resize the images to a standard dimension of 128 × 128 × 128 pixels. Normalization of the mean and standard deviation was then used to ensure consistency in the scale of the imaging data. In unimodal modeling, tabular and CT image data were used as separate inputs. In the multimodal model, features from both modalities were fused at the feature level and used as a combined input.

### Unimodal model selection and validation

In this study, fivefold cross-validation was employed for training and validating all tabular and CT data. Based on the existing test sets, the training and validation sets were partitioned at an 8:2 ratio. Table Transformer and SwinTransformerForClassification (used as feature extractors) were utilized to extract features from tabular and CT image data, respectively. The core concept of Table Transformer is to use a Transformer architecture based on self-attention mechanisms to convert the embeddings of categorical features into robust contextual embeddings, which exhibit high tolerance to missing and noisy data features. The Swin Transformer backbone maps CT images into an embedding sequence through a 3D-to-1D projection method and computes self-attention mechanisms using shifted windows to extract features at five different resolutions. The Swin Transformer architecture has demonstrated superior capability in extracting semantic features from CT images.

### Analysis of disease progression risk in patients without primary resistance

To further explore the utility of the model, we assessed its ability to predict disease progression risk among patients without primary resistance (the acquired resistance group, class 0). For this analysis, patients in the acquired resistance group within each test cohort (Test Cohorts 1 and 2) were stratified into two equal-sized subgroups (high and low risk) based on the median of their MC-Trans-predicted primary resistance probabilities. Prognostic differences between these subgroups were then compared using Kaplan–Meier survival curves and the log-rank test. Hazard ratios (HRs) and 95% confidence intervals (CIs) were calculated to quantify the difference in progression risk.

### Expert panel prediction

The expert panel involved in this study consisted of three medical oncologists, each with more than 15 years of clinical experience. None of the participants were involved in the treatment of the study cases, and they were blinded to the proportion of patients with primary resistance in the dataset. To ensure a fair comparison with the MC-Trans model, the expert panel was granted access to the complete dataset for each patient in Test Cohort 2. This included the full set of 98 tabular features (comprising general information, treatment history, NGS genomic profiling, and laboratory tests) and the corresponding baseline CT images (DCM format). Each physician was instructed to leverage their clinical expertise to holistically synthesize all the provided multisource data and independently assessed the likelihood of primary resistance for each case, assigning a score from 1 (least likely) to 10 (most likely). The scores from the three experts for each patient were summed and divided by the total possible score of 30 to obtain the expert panel’s predicted probability of primary resistance for that patient through Eq. 1. The Eq. 1 is as follows: Let S_ij_ represent the primary resistance likelihood score (where *j* = 1,2,3; score range 1–10) assigned by the j-th expert to the *i*-th patient. Let P_i_ represent the probability of primary resistance for the i-th patient as predicted by the expert panel.

1 $${{\rm{P}}}_{{\rm{i}}}=({{\rm{S}}}_{{\rm{i}}1}+{{\rm{S}}}_{{\rm{i}}2}+{{\rm{S}}}_{{\rm{i}}3})/30$$

### Ethical approval

This study has passed the ethical review. The consent letters for ethical review are: 2025-063-01 and KY-20250604001-01.

### Statistical analysis

Model performance was evaluated using accuracy, precision, recall, F1 score, ROC-AUC, and PR-AUC on the test sets. To assess the significance of differences in the ROC-AUC and PR-AUC, the bootstrap resampling method (1000 resamples) was employed, from which 95% confidence intervals (CIs) for the differences and P values were calculated. Prognostic differences were compared using Kaplan–Meier survival curves. Statistical analyses were performed in Python version 3.9.0. The graphs were generated using GraphPad Prism version 10.1.2. Deep learning models were constructed using PyTorch version 2.4.0. A two-sided *P* < 0.05 was considered to indicate statistical significance.

## Data Availability

The patient-level data generated in this retrospective study are protected patient health information and are not publicly available due to privacy and ethical restrictions imposed by the Ethics Committees of The Affiliated Yixing Hospital of Jiangsu University and Lianyungang Clinical College of Nanjing Medical University. De-identified data may be made available to qualified researchers for the purpose of replicating the study findings, upon reasonable request to the corresponding author and with approval from the aforementioned Ethics Committees. Requests will be evaluated based on scientific merit and data use agreement compliance.

## Code availability

The implementation of the MC-Trans deep learning model and the scripts for data preprocessing, training, and evaluation are publicly available on GitHub at https://github.com/MC-Trans/MC-Trans.
